# Progress in Disease of Digestive Organs

**Published:** 1903-12-12

**Authors:** 


### Progress in Disease of Digestive Organs.

(*Concluded from page* 170.)

**Diseases of the Liver and Gall-bladder.**—The differential diagnosis of the various causes of jaundice has been dealt with by Phillips,[35] who considers that the term "obstructive" may be retained as denoting obstruction of the common duct, though it has been shown that intrahepatic causes of jaundice are in their nature also obstructive. Thus Weber[39] describes a biliary cirrhosis dependent on the obstruction of the larger ducts by calculi (12 cases), and Anders[40] classes as "toxic" the jaundice depending on æther narcosis, syphilis, and bacillary infection (as from cholecystitis). He shows that in such cases there is probably some blocking of the liver ducts from swelling of the cells and that excessive blood destruction occurs, leading to a viscid condition of the bile favourable to obstruction and consequent jaundice. Among rare conditions Rolleston[39] cites instances of the condition known as "simple family cholæmia," to which a variety of secondary symptoms have been attributed, but which is sometimes compatible with health. He also alludes to cases of recurrent jaundice associated with enlarged spleen and occurring in families, probably unconnected with Hanot's disease; and quotes 14 cases of icterus neonatorum occurring in one family with only four survivors. The treatment of biliary cirrhosis by draining the gall-bladder to get rid of bacterial infection has been tried in 17 cases (Greenough[41]), of which 13 have improved; some cases were doubtful, but most appeared to be of the type described by Hanot. Cirrhosis of the ordinary alcoholic form with ascites has been treated by operation in 122 cases (Greenough), 44 of which were improved. The object is to relieve ascites by increasing the vascular anastomoses through artificial adhesions, but it appears only useful where the ascites is due to chronic peritonitis (Thomson, Hale White).[42] Fisher[43] has collected seven cases of *thrombosis of the hepatic veins*, characterised by ascites, dilatation of the abdominal veins, and sometimes gastro-intestinal hæmorrhages; Moore,[44] who collected 12 cases, found hæmatemesis rare. The ætiogy is very obscure, but it is probably not syphilitic. *Primary sarcoma* of the liver may be distinguished from carcinoma by its even more rapid course, the larger size of the tumour and the infrequency of ascites and jaundice (Pepere.)[39] *Chronic perihepatitis* may be considered clinically as falling into two groups (Nicholls)[51]: (1) Primary perihepatitis with ascites and enlarged liver and spleen with subsequent pleurisy and pericarditis, (2) primary pericarditis with adherent pericardium and mediastinitis. In both forms ascites is prominent and jaundice and hæmorrhages rare. Pick believes the ascites to be due to liver changes, but Kelly, in 31 cases out of 39, found chronic peritonitis or perihepatitis, which he considered responsible.[39] The gall bladder is regarded by Woods Hutchinson[45] and Gibbon[40] as a vestigial structure of practically no functional importance and low resistance to disease, very comparable in this respect to the appendix cæci. When gangrenous, immediate operation is necessary, and though the differential diagnosis may not be clear at first, it is usually easy under anæsthesia (Gibbon). Short of this, stagnation of the bile and bacterial invasion produce gall-stones and this by a second invasion leads to cholecystitis (Stewart).[33] Slight attacks are often regarded as gastric in origin, but the diagnosis may be made by the tenderness over the gall-bladder during or after a seizure and their want of relationship to the ingestion of food. Various micro-organisms are found in these cases, notably the typhoid and colon bacilli, the latter also giving rise to hepatic abscess (Sheldon).[40] Both the last-named authors think that under favourable circumstances the colon bacillus may become pathogenic. If the organisms are highly virulent, empyema of the gall-bladder may occur. Adhesions dragging on or dilating the stomach, cancer, cirrhosis, and pancreatic disease are remote effects. Stewart states that no medication will dissolve gall-stones, and that olive oil, which does good in some cases, acts by forming glycerine in the duodenum and thus promoting peristalsis. Treatment, therefore, must be directed towards decreasing inflammatory processes when started, and thus obviating the remote results. A stay at Carlsbad is very desirable, if possible. *Liver abscess* rarely results from infection by the bile passages and is generally viâ the portal vein (Sheldon). For this reason the abscesses are usually multiple and the liver enlarged diffusely. After typhoid, infection by the hepatic artery occurs occasionally and usually in conjunction with abscesses in other parts. The symptoms are general and local (pain, enlargement, and tenderness over the liver), but the latter group may be absent (Dunin and Osler).[46] The treatment is surgical even, according to Sheldon, when the abscesses are multiple.

**Animal Parasites.**—Attention has been recently given to the intestinal parasites imported into the United States from Cuba, the Philippines and Porto Rico. Stiles has distinguished two varieties of ankylostoma found in man; one known as the Old World hookworm and the other as the New, or Uncinaria Americana. Brooks[46] and Smith[47] have also studied this parasite; the latter observer found it in eight cases among 88 healthy persons. Smith drew attention to a similar organism U. stenocephala, common in dogs, but Brooks is of opinion that intestinal parasites are seldom transmitted from one animal to another, as each species has its special variety. Clayton[47] has experimented on dogs as to the safety of thymol which he considers an effective parasiticide. It must, however, be given with caution, 2–4 grams in a dry powder being safe for man, followed by an ounce or so of brandy, and in a few hours by a purge. Stronguloides intestinalis is rare in temperate climates, but causes in Cochin China a peculiar form of diarrhœa (Price).[47] In Scandinavia a form of dysentery resembling the amœbic is caused by Balantidium coli. This worm though recognised by Malmesden in animals, has only recently been proved by Brooks and Strong to produce the disease in man. Dévé ([48]) has collected 15 cases of rupture of hydatid cyst into the peritoneum; no sufficient cause for the accident was found, and the entrance of bile into the cyst was a subsequent event. No jaundice occurs when bile gets into the peritoneum, but ascites is troublesome. Secondary infection is not prevented by the presence of bile, and occasionally the hooklets set up a condition resembling miliary tuberculosis.

[39] Pract. Mar., 1903. [40] Amer. Jour. Med. Sci., April, 1903. [33] Ibid., Mav, 1903. [41] Ibid., vol. cxxiv., p. 979. [42] Pract. Mar., 1903, p. 374. [43] Bristol Med. Chi. Jour., Sept., 1902. [44] Med. Chron., July, 1902. [45] Med. Rec., 1903, i., p. 770. [46] Ibid., p. 472. [47] Ibid., p. 757. [48] Pract. Mar., 1903, p. 380. [51] Pract. Mar. 1903, p. 382.

